# *Sedum aizoon* L.: a review of its history, traditional uses, nutritional value, botany, phytochemistry, pharmacology, toxicology, and quality control

**DOI:** 10.3389/fphar.2024.1349032

**Published:** 2024-03-14

**Authors:** Bai-Ling Wang, Zhen-Kai Ge, Jing-Ran Qiu, Si-Qi Luan, Xin-Cai Hao, Yong-Heng Zhao

**Affiliations:** ^1^ School of Pharmaceutical Sciences, Hubei University of Medicine, Shiyan, China; ^2^ Hubei Key Laboratory of Wudang Local Chinese Medicine Research, Shiyan, China; ^3^ Hubei Provincial Technology and Research Center for Comprehensive Development of Medicinal Herbs, Shiyan, China

**Keywords:** *Sedum aizoon* L., pharmacological activities, quality control, hemostatic activity, active metabolites

## Abstract

In China, Russia, Mongolia, Japan, North Korea, and Mexico, *Sedum aizoon* L. (*S. aizoon*) is used as an edible plant. Up to now, over 234 metabolites, including phenolic acids, flavonoids, triterpenes, phytosterols, and alkaloids, among others, have been identified. In addition to its antioxidant, anti-inflammatory, anti-fatigue, antimicrobial, anti-cancer, and hemostatic activities, *S. aizoon* is used for the treatment of cardiovascular disease. This paper provides an overview of the history, botany, nutritional value, traditional use, phytochemistry, pharmacology, toxicology, and quality control of *S. aizoon*.

## Graphical Abstract

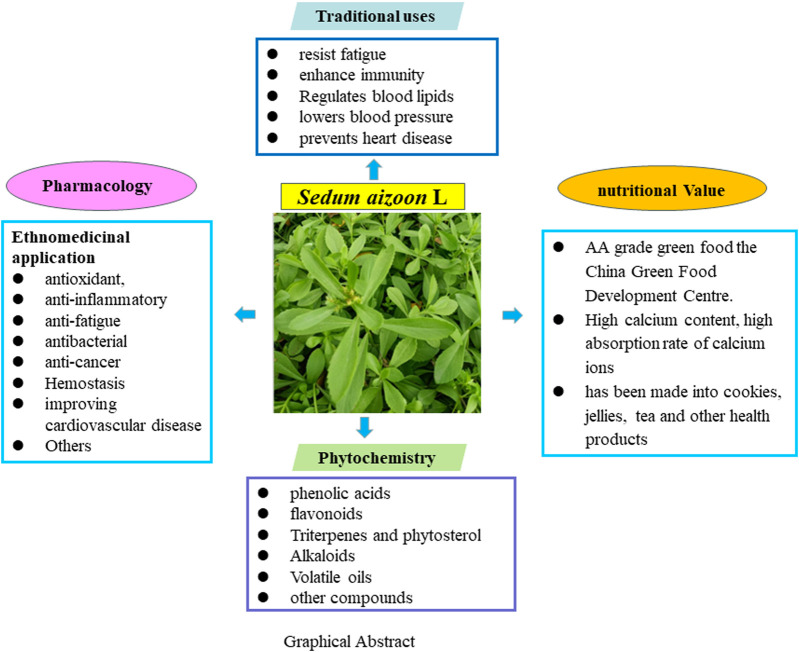

## Highlights

• *S. aizoon* L. is frequently prescribed in both China and other countries as a traditional folk herbal remedy for various diseases
*•* This review contributes to updating the herbalogical textual research, traditional use, botany, phytochemistry, pharmacology, toxicity, and nutritional value and quality control of *S. aizoon* L.
*•* In earlier literature, there was no systematic review of *S. aizoon* L

## 1 Introduction

*Sedum aizoon* L. (Chinese name:景天三七) is a perennial herbaceous plant that is widely distributed in China, Russia, Mongolia, Japan, North Korea, and Mexico. It is a member of the *Sedum* genus in the *Sedum* family (*Crassulaceae*) (Guo and Lin, 2007). Its name is also consistent with the plant name recorded in “The Plant List” (http://www.theplantlist.org), which is now incorporated into the requirement for traditional medicine in the provinces of Jiangsu and Fujian (Jia et al., 2014). It is one of the renowned “Taibai seven medicine (太白七药)” in the Qinling Mountains, which has the effects of dispersing blood stasis, stopping bleeding, tranquilizing the mind, detoxifying, and analgesia, and is used in the treatment of various kinds of bleeding, palpitations, and insomnia. Growing in the natural environment, *S. aizoon* is a unique pest-free plant that does not require pesticides during its whole phenological cycle and has been designated as AA grade green food by the China Green Food Development Center. Its fresh stems and leaves are consumed as vegetables (Xue, 2015).

Despite the fact that the phytochemistry and ethnopharmacology of *S. aizoon* have been previously reviewed, a comprehensive study linking its bioactive metabolites with its pharmacological properties is lacking. Therefore, this paper provides an overview of the history, botany, nutritional value, traditional use, phytochemistry, pharmacology, toxicology, and quality control of *S. aizoon*.

## 2 Materials and methods

Information about *S. aizoon* was gathered from scientific literature sources, including PubMed, Baidu Scholar, Google Scholar, Web of Science, SciFinder, CNKI, Wanfang, the Plant List (www.theplantlist.org), and books. The history, nutritional value, traditional uses, botany, phytochemistry, pharmacology, toxicology, and quality control or a combination between them was used as keywords to search for data up to July 2023. Approximately, 767 research studies of *S. aizoon* were gathered from various databases. With the removal of duplicate literatures, 300 literatures were selected according to research purpose, relevance, and article type. The articles which contained information apart from that mentioned above or written in languages rather than English were also excluded. ChemBioDraw Ultra version 14.0 was used to draw chemical structures.

## 3 History and traditional uses

### 3.1 History

*S. aizoon* was first recorded in “*Jiu Huang Ben cao*” (救荒本草) (Ming Dynasty), which is the earliest book with agronomy and botany as its monograph on the history of China. Later, it was also included in many other famous works on Chinese herbal medicine, including “*Wild Vegetables Bo lu*” (野菜博录) (Ming Dynasty), “*Plants Ming Shi Tu Kao*” (植物名实图考), and “*Discussion on varieties of Chinese medicinal materials*” (中药材品种论述).

The medicinal parts of *S. aizoon* were roots and grass in *S. aizoon*, and *S. kamshaticum*. *S. aizoon* has more than 60 synonyms and is distributed in more than 20 provinces or autonomous regions. In addition, the herb and the syrup were included in the Pharmacopoeia of the People’s Republic of China (Part I) (1977 edition) (Chinese Pharmacopoeia Committee, 2005).

### 3.2 Traditional uses

In folk medicine, the flat and sweet whole herb and the roots of *S. aizoon* are widely used for dispersing blood stasis and stopping blood bleeding. For instance, daily administration of 60–90 g of *S. aizoon* decoction can treat bleeding symptoms, including hemoptysis, bleeding gums, epistaxis, gingival bleeding, and internal bleeding. The fresh juice was effectively used for the treatment of leukemia, aplastic anemia, thrombocytopenic purpura, hemoptysis, and different forms of bleeding (i.e., gingival, digestive tract, and hematuria) (Chinese herbal medicine research group, 1971). In addition, ancient medical classics, such as Li Shizhen’s “Compendium of Materia Medica” (本草纲目), Chen Shiduo’s “New Compilation of Materia Medica” (本草新编), and Zhang Xichun’s “Intergrating Chinese And Western Medicine” (医学衷中参西录), explicitly stated that *S. aizoon* had good hemostasis and analgesic function, which was known as “the god medicine for hemostasis” (止血神药). It is also used as a heart and mind tranquillizing agent with an excellent effect on hysteria palpitation, restlessness, hypertension, and rheumatic heart disease (Chen, 2003). Likewise, the detoxifying and clearing heat effects have also been reported.

Of note, *S. aizoon* has a long history as both an edible and medicinal herb. For example, vegetables with *S. aizoon*’s stems and leaves as metabolites have good nutritional value. “Jiu Huang Ben Cao” (救荒本草) in the Ming Dynasty stated that the regular consumption of the fresh, tender stems and leaves of *S. aizoon* can promote blood circulation and calm the heart.

## 4 Nutritional value

The tender stems and leaves contain moisture (87 g), protein (2.1 g), fat (0.7 g), carbohydrate (8.0 g), crude fiber (1.5 g), ash (1.2 g), energy (196.65 KJ), Ca (315 mg), P (39 mg), Fe (3.2 mg), carotene (2.54 mg), vitamin B1 (0.05 mg), vitamin B2 (0.07 mg), vitamin PP (90 mg), and vitamin C (90 mg) (Yi, 2000; Liu et al., 2012). Owing to its unique aroma and taste, *S. aizoon* is used for the preparation of cookies, jellies, and tea (Wang, 2013).

## 5 Botany

### 5.1 Geographical repartition

*S. aizoon* belongs to the genus *Sedum* of the *Crassulaceae* family. There are approximately 600 species widely distributed in the temperate and subtropical regions of the northern hemisphere with Mexico being the largest center of origin and diversity of *Sedum* species.

### 5.2 Morphology

**FIGURE 1:**
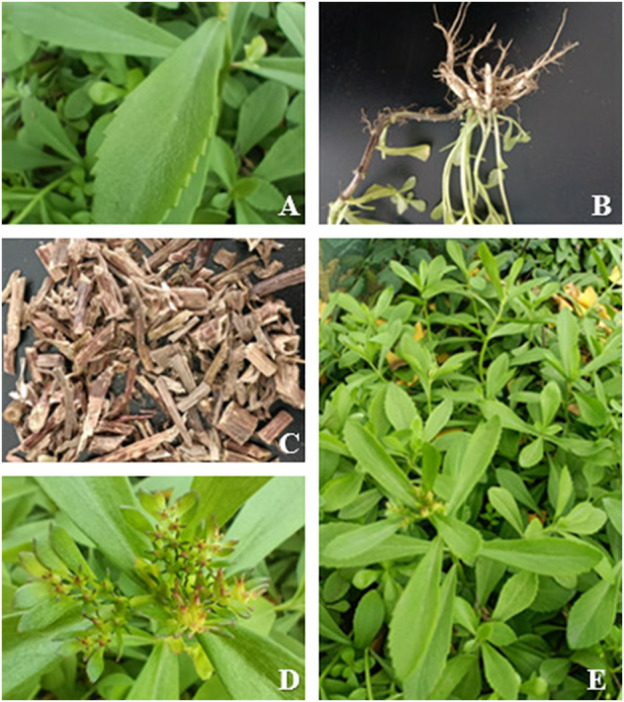
Morphological characteristics of *S. aizoon*: **(A)** leaves, **(B)** roots, **(C)** dry drug, **(D)** buds, and **(E)** whole plant.

*S. aizoon* is an annual or perennial, succulent herb, growing in clusters and has a strong ability to bifurcate. *S. aizoon* has coarse, woody rhizomes that resemble ginseng in form. The stems are erect, cylindrical, and glabrous, which can reach heights of 15–50 cm. At each node, the stems carry just one leaf, which is nearly opposite on both sides. The leaves are 2.5–5 cm long, 5–12 mm wide, obovate or long oval in shape, and broad and thick with more juice. Additionally, they feature a cuneate base, a serrated border toward the apex, a moderately rounded top, and few sessile leaves. The loose, terminal verticillaster contains ten stamens that are around the same length as the petals, five distinct pistils that are slightly longer than the stamens, five orange–yellow petals with lancolate, sharp tips, and five sepals with blunt ends. Follicles are either reddish or brown in color and are grouped in a star pattern. Seeds are obovate, smooth, have wings along the edge, and have a wider apical. Flowers usually bloom in summer. The photos of *S. aizoon* are pictured and shown in Figure 1.

## 6 Phytochemistry

**FIGURE 2:**
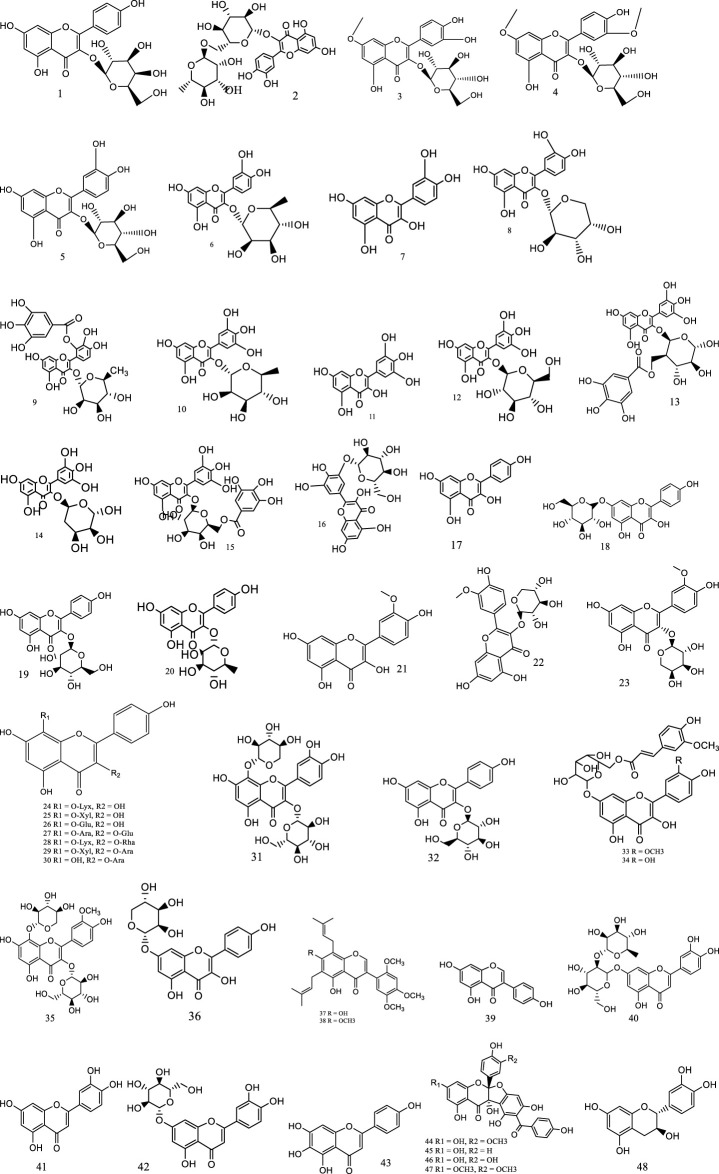
Structures of flavonoids from *S. aizoon* (**1–48**).

**FIGURE 3:**
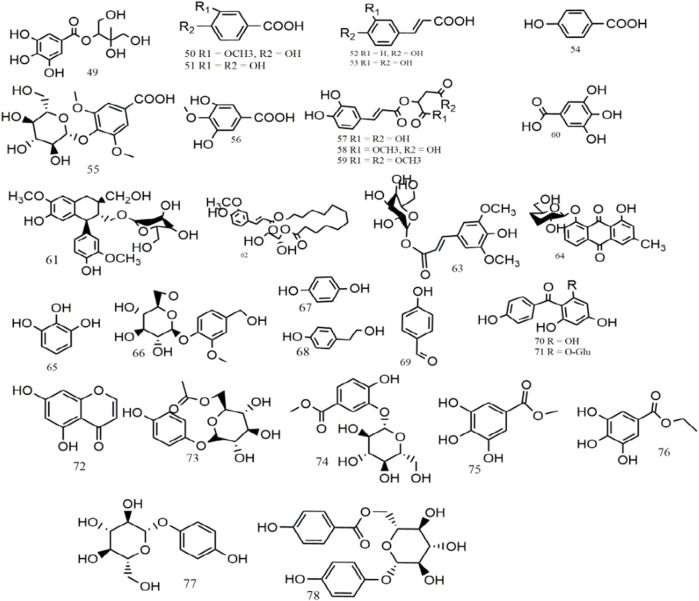
Structures of phenolic acids from *S. aizoon* (**49–78**).

**FIGURE 4:**
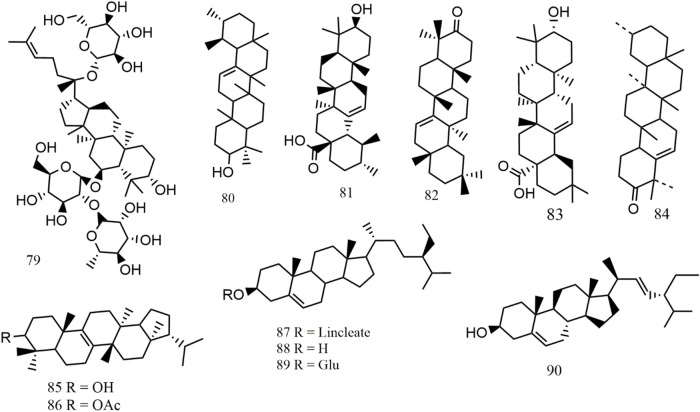
Structures of triterpenoids (**79–86**) and phytosterol (**87–90**) from *S. aizoon*.

**FIGURE 5:**
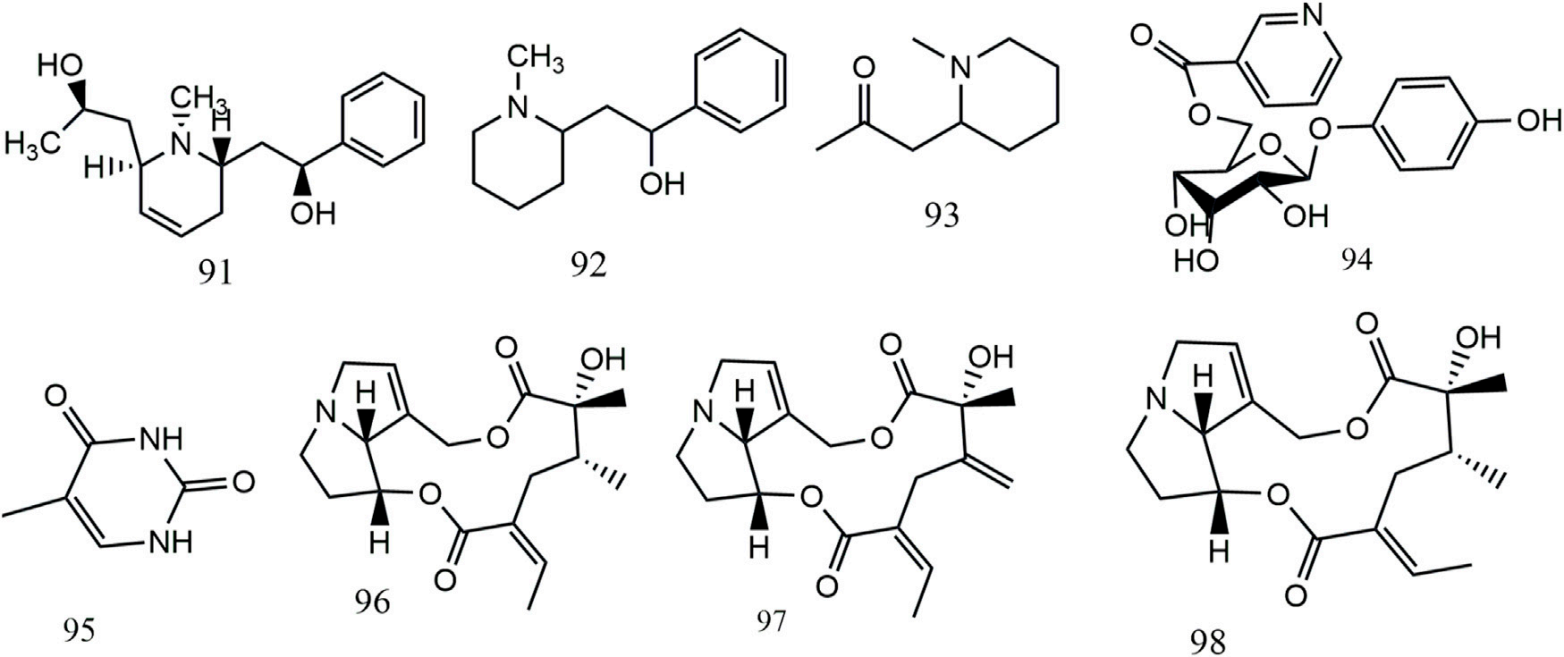
Structures of alkaloids (**91–98**) from *S. aizoon*.

Up to now, more than 234 metabolites, including flavonoids (**1–48**), phenolic acids (**49–78**), triterpenes and phytosterols (**79–90**), alkaloids (**91–98**), volatile constituents (**99–216**), and others (**217–234**), have been preliminarily isolated or identified from *S. aizoon*. Among these, flavonoids are the main metabolites of *S. aizoon*. The main metabolites and their structure are given in Table 1 and Figures 2–5.

**TABLE 1:** Main active metabolites identified in *S. aizoon*.

Metabolite	Plant part	Molecular formula	Reference
Flavonoid			
Trifolin	Leaves and stems	C_21_H_20_O_11_	Xu et al. (2019)
Rutin		C_27_H_30_O_16_	
Isoquercitrin		C_21_H_20_O_12_	
Isorhamnetin		C_16_H_12_O_7_	
Astragalin		C_21_H_20_O_11_	
Genistein		C_15_H_10_O_5_	
Lonicerin		C_27_H_30_O_15_	
Scutellarein		C_15_H_10_O_6_	
Catechin		C_15_H_14_O_6_	
Rhamnetin-3-O-β-D-glucopyranoside	Rhizome	C_22_H_22_O_12_	Li et al. (2020a)
Isorhamnetin-3-O-β-D-xylopyranoside		C_21_H_20_O_11_	
Isorhamnetin-3-O-α-L-arabinopyranoside		C_21_H_20_O_11_	
Rhamnazin-3-O-β-D-glucopyranoside	Aerial parts	C_23_H_26_O_12_	Xiong et al. (2019)
Quercetin	Aerial parts, rhizome, and leaves and stems	C_15_H_10_O_7_	
Myricetin	Aerial parts and leaves and stems	C_15_H_10_O_8_	
Luteoloside	N/A	C_21_H_20_O_11_	
Quercitrin	Aerial parts and leaves and stems	C_21_H_20_O_11_	Wolbi and Olszewska (1996), Li et al. (2007)
Myricitrin	Aerial parts	C_21_H_20_O_12_	
Quercetin-3-o-(2′-galloyl) rhamnoside	N/A	C_28_H_30_O_9_	Wolbi and Olszewska (1996)
Quercetin-3-O-α-L-arabinopyranoside	Leaves and stems and rhizome	C_20_H_18_O_11_	Han et al. (2017)
Myricetin-3-O-α-L-arabinopyranoside	Aerial parts	C_20_H_18_O_12_	
Kaempferol-7-O-glucoside	Leaves and stems	C_21_H_20_O_11_	
Kaempferol-3-O-β-D-glucopyranoside		C_21_H_20_O_11_	
Herbacetin-3-O-α-L-arabinopyranoside		C_20_H_18_O_10_	
Myricetin-3-β-D-glucopyranoside	Aerial parts and leaves and stems	C_21_H_20_O_13_	Li et al. (2008)
Myricetin-3-β-D-(6″-o-galloyl)-glucopyranoside	Whole grass	C_28_H_24_O_17_	
Myricetin-3-o-β-D-(6″-o-galloyl)-galactopyranoside		C_28_H_24_O_17_	
Myricetin-3′-o-β-D-glucopyranoside	Leaves and stems	C_21_H_20_O_13_	Jia et al. (2014)
Kaempferol	Leaves and stems and rhizome	C_15_H_10_O_6_	Lin et al. (2014), Xiong et al. (2019)
Kaempferol-3-O-α-L-rhamnoside	Leaves and stems	C_21_H_20_O_10_	Zhang et al. (2010)
Herbacetin-8-O-α-D-lyxoside		C_20_H_18_O_11_	
Herbacetin-8-O-β-D-xylopyranoside		C_20_H_18_O_11_	
Luteolin		C_15_H_10_O_6_	
Herbacetin-8-O-β-D-glucopyranoside	Aerial parts	C_25_H_23_O_7_D_3_	Xu et al. (2015)
Herbacetin-3-O-β-D-glucopyranosyl-8-O-α-L-arabinopyranoside		C_74_H_105_O_32_	
Herbacetin-3-O-α-L-rhamnopyranosyl-8-O-α-D-lyxopyranoside		C_26_H_28_O_14_	
Herbacetin-3-O-α-L-arabinopyranosyl-8-O-β-D-xylopyranoside		C_25_H_26_O_14_	
Gossypetin-3-O-β-D-glucopyranosyl-8-O-β-D-xylopyranoside		C_73_H_106_O_34_	
3′-Methoxyl-gossypetin-3-O-β-D-glucopyranosyl-8-O-β-D-xylopyranosie		C_27_H_30_O_17_	
6″-O-(E)-feruloyl isorhamnetin	Whole plant	C_32_H_30_O_15_	(Li J. X. et al., 2011)
6″-O-(E)-feruloyl quercetin		C_31_H_28_O_15_	
3,4′,5,7-Tetrahydroxyflavone-7-O-α-D-xylopyranoside	Whole grass	C_20_H_18_O_10_	Han et al. (2021)
Sedacin A	Whole plant	C_28_H_32_O_7_	Li J. X. et al. (2011)
Sedacin B		C_29_H_34_O_7_	
1,3,8,10,10b-Pentahydroxy-5a-(4-hydroxy-3-methoxyphenyl)-9-(4-hydroxybenzoyl)-5a,10b-dihydro-11H-benzofuro[2,3-b]chromen-11-one	Roots	C_29_H_21_O_12_	Li et al. (2017)
1,3,8,10,10b-Pentahydroxy-9-(4-hydroxybenzoyl)-5a-(4-hydroxyphenyl)-5a,10b-dihydro-11H-benzofurochromen-11-one		C_28_H_19_O_11_	
5a-(3,4-Dihydroxyphenyl)-1,3,8,10,10b-pentahydroxy-9-(4-hydroxybenzoyl)-5a,10b-dihydro-11H-benzofurochromen-11-one		C28H19O12	
1,8,10,10b-Tetrahydroxy-5a-(4-hydroxy-3-methoxyphenyl)-9-(4-hydroxybenzoyl)-3-methoxy-5a,10b-dihydro-11H-benzofuro[2,3-b]chromen-11-one		C_30_H_23_O_12_	
Phenolic acids			
Sedumol	Whole grass	C_12_H_16_O_8_	Han et al. (2021)
Vanillic acid	Aerial parts	C_8_H_8_O_4_	Lin (2014)
Protocatechuic acid	Aerial parts and leaves and stems	C_7_H_6_O_4_	
Caffeic acid	N/A	C_9_H_8_O_4_	
P-hydroxybenzoic acid	Aerial parts and leaves and stems	C_7_H_6_O_3_	Lin et al. (2014)
Pyrogallol	Aerial parts	C_6_H_6_O_3_	
5,7-Dihydroxychromone	N/A	C_9_H_6_O_4_	
Glucosyringic acid	Leaves and stems	C_15_H_20_O_10_	Jia et al. (2014)
P-hydroxybenzoyl arbutin		C_19_H_20_O_9_	
Pyroside		C_14_H_18_O_8_	
Arbutin	Roots and leaves and stems	C_12_H_16_O_7_	
4-Methoxy-3,5-dihydroxybenzoic acid	Whole grass	C_8_H_8_O_5_	Han et al. (2021)
4-Hydroxybenzeneethanol		C_8_H_10_O_2_	
4-Hydroxybenzaldehyde		C_7_H_6_O_2_	
cis-4-Coumaric acid	Aerial parts	C_9_H_8_O_3_	Xiong et al. (2019)
2-O-(trans-caffeoyl) malic acid		C_13_H_12_O_8_	
2-O-(trans-caffeoyl)-malic acid 1-methyl-ester		C_14_H_14_O_8_	
2-O-(trans-caffeoyl)-malic acid 1,4-dimethyl ester		C_15_H_16_O_8_	
Isolariciresinol-9-O-β-D-glucopyranoside		C_26_H_34_O_11_	
Iriflophenone-2-O-β-D-glucopyranoside		C_19_H_20_O_10_	
Ethyl gallate	Aerial parts and leaves and stems	C_9_H_10_O_5_	
Gallic acid	Aerial parts, whole plant, and leaves and stems	C_7_H_6_O_5_	Zhang et al. (2010)
Methyl gallate	Aerial parts and leaves and stems	C_8_H_8_O_5_	
Echinochlorin A	Rhizome	C_26_H_40_O_8_	Li et al. (2020a)
1-O-sinapoyl glucopyranoside	Aerial parts	C_17_H_22_O_10_	Xu et al. (2015)
Chrysophanol-8-O-β-D-glucoside	Whole grass	C_21_H_20_O_9_	Li et al. (2008)
Hydroquinone	Roots and whole grass	C_6_H_6_O_2_	
Vanilloloside	Leaves and stems	C_14_H_20_O_8_	Han et al. (2017)
Woodorien		C_13_H_9_N_3_O_2_	
Iriflophene	Aerial parts and rhizome	C_13_H_10_O_5_	Xiong et al. (2019), Li et al. (2020a)
Triterpenes			
Ginsenoside Re	Roots	C_48_H_82_O_18_	Gong (2020)
α-Amyrin	N/A	C_30_H_50_O	
Ursolic acid	Roots	C_30_H_48_O_3_	Li et al. (2008)
Glutin-5-en-3-one	Leaves and stems	C_30_H_48_O	
Isomoliol-3β-acetate		C_32_H_52_O_2_	
Taraxerone	Rhizome	C_30_H_48_O	Li et al. (2020a)
Isomotiol		C_30_H_50_O	
Oleanolic acid	Roots	C_30_H_48_O_3_	Lin (2014)
Phytosterols			
β-Sitosteryl linoleate	Rhizome	C_47_H_80_O_2_	Li et al. (2020a)
Daucosterol	Rhizome and whole grass	C_35_H_60_O_6_	
β-Sitosterol	Rhizome, leaves and stems, and roots	C_29_H_50_O	Zhang et al. (2010)
Stigmasterol	N/A	C_29_H_48_O	Cao (2011)
Alkaloids			
Sedinine	N/A	C_17_H_25_NO_2_	Kim et al. (1996)
Despun methylisopelletierine		C_9_H_17_NO	
Sedamine	Roots	C_14_H_21_NO	Li et al. (2008)
Aizoonoside A	Aerial parts	C_18_H_19_NO_8_	Xu et al. (2015)
Thymine	Aerial parts	C_5_H_6_N_2_O_2_	Lin et al. (2014)
Senecionine	Roots	C_18_H_25_NO_5_	Wu et al. (2008)
Seneciphylline		C_18_H_23_NO_5_	
Integerrimine		C_18_H_25_NO_5_	
Volatile oils			
2,6-Di(tbutyl)-4-hydroxy-4-methyl-2,5-cyclohexadien-1-one	Whole plant	C_15_H_24_O_2_	Qian et al. (2018)
β-Ionone		C_13_H_20_O	
Epiglobulol		C_15_H_26_O	
α-Guaiene		C_15_H_24_	
Isophytol		C_20_H_40_O	
Squalene		C_30_H_50_	
Tritriacontane		C_33_H_68_	
Hexadecane		C_16_H_34_	
Pristane		C_19_H_40_	
Octadecane		C_18_H_38_	
Tricosane		C_23_H_48_	
Tetracosane		C_24_H_50_	
Pentacosane		C_25_H_52_	
Hexacosane		C_26_H_54_	
Heptacosane		C_27_H_56_	
Octacosane		C_28_H_58_	
Nonacosane		C_29_H_60_	
Hentriacontane		C31H64	
Cetyl palmitate		C_32_H_64_O_2_	
4, 8, 12, 16-Tetramethyl heptadecan-4-olide		C_21_H_40_O_2_	
Cyclohexyl benzoate		C_13_H_16_O_2_	
Methyl palmitoleate		C_17_H_32_O_2_	
Methyl palmitate		C17H_34_O_2_	
Ethyl palmitate		C_18_H_36_O_2_	
Methyl linolelaidate		C19H34O2	
Methyl oleate		C_19_H_36_O_2_	
Methyl stearate		C_19_H_38_O_2_	
Ethyl linoleate		C_20_H_36_O_2_	
Ethyl oleate		C_20_H_38_O_2_	
1-Hexacosanol		C_26_H_52_O	
Hexahydrofarnesyl acetone	Whole plant and fresh herbs	C_18_H_36_O	Guo et al. (2006), Qian et al. (2018)
2-Undecanone	Fresh herbs	C_11_H_22_O	Guo et al. (2006)
2-Tridecanone		C_13_H_26_O	
Nerolidol		C_15_H_26_O	
(−)-Spathulenol		C_15_H_24_O	
Cedrol		C_15_H_26_O	
Globulol		C_15_H_26_O	
1-Nonene		C9H18	
(十)-Aromadendrene		C15H24	
Calamenene		C_15_H_22_	
Caryophyllene epoxide		C_15_H_24_	
Bornyl acetate		C_12_H_20_O_2_	
Geraniol acetate		C_12_H_20_O_2_	
15-ene-heptadecanal		C_17_H_48_O	
Hexadecanoic acid		C_16_H_32_O_2_	
Phytol	Leaves, stems, fruits, and fresh herbs	C_20_H_40_O	
4-hepten-2-one	Aerial parts	C_7_H_12_O	Chen et al. (2014)
Elsholtzia ketone		C_10_H_14_O_2_	
3-Methyl-2-butanol		C_5_H_12_O	
2,3-Butanediol		C_4_H_10_O_2_	
1-Octanol		C_8_H_18_O	
4-Terpineol		C_10_H_18_O	
3-Hexen-1-ol		C_6_H_12_O	
Pentylfuran		C_9_H_14_O	
β-Phellandrene		C_10_H_16_	
4-Carene		C_10_H_16_	
β-Terpinene		C_10_H_16_	
Isoterpinolene		C_10_H_16_	
α-Thujene		C_10_H_16_	
β-Farnesene		C_15_H_24_	
π-Muurolene		C_15_H_24_	
Heptanal		C_7_H_14_O	
Benzaldehyde		C_7_H_6_O	
Hexanal		C_6_H_12_O	
Furfural		C_5_H_4_O_2_	
Octanal		C_8_H_16_O	
Benzeneacetaldehyde		C_8_H_8_O	
Nonanal		C_9_H_18_O	
Decanal		C_10_H_20_O	
1-Octadecanol	Roots and leaves	C_18_H_38_O	Chen and Qiang (2017)
(Z) 9-Octadecenoic acid, methyl ester	Roots and stems	C_19_H_36_O_2_	
2,2′-Methylenebis(6-tert-butyl-4-methylphenol	Leaves, stems, and fruits	C_23_H_32_O2	
Dimethyl phthalate		C_10_H_10_O_4_	
Methyl tetradecanoate		C_15_H_30_O_2_	
Heptadecanoic acid methyl ester		C_18_H_36_O_2_	
Pentatriacontane	Leaves, stems, and roots	C_35_H_72_	
Heptadecane	Leaves, and whole plant	C_17_H_36_	
3-Ethyl-2,4-dimethyl-pentane	Leaves	C_9_H_20_	
2,6-Dimethyl-octane		C_10_H_22_	
6,10,14-Trimethyl2 pentadecanone		C_18_H_36_O	
1-Pentadecanol		C_15_H_32_O	
Oxacycloheptadec-8-en-2-one		C_16_H_28_O_2_	
Tridecanoic acid, methyl ester		C_14_H_28_O_2_	
2,6,11-Trimethylodlodecane		C_15_H_32_	
3-Methyl-undecane		C_12_H_26_	
Octadecane	Fruits	C_18_H_38_	
2,6,10,14-Tetramethyl-hexadecane		C_20_H_42_	
Icosane	Stems	C_20_H_42_	
Nonadecane		C_19_H_40_	
3,8-Dimethyl-decane		C_12_H_26_	
4-Methyl-pentadecane		C_16_H_34_	
1-Octadecene		C_18_H_36_	
2-Methyl-tridecane		C_14_H_30_	
Tetratetracontane		C_44_H_90_	
Tetradecane		C_14_H_30_	
Pentadecane	Leaves and stems	C_15_H_32_	
2,4,4-Trimethylhexane		C_9_H_20_	
2,4-Dimethylhexane		C8H18	
4,6-Dimethyl-dodecane		C_14_H_30_	
Heneicosanoic acid-methyl ester		C_22_H_44_O_2_	
Tricosanoic acid, methyl ester		C_24_H_48_O_2_	
2,4-bis(1,1-Dimethylethyl)-phenol		C_14_H_22_O	
Hexadecyl-oxirane		C_18_H_36_O	
3,3- Dimethylhexane		C_8_H_18_	
3, 3-Dimethyl-heptane	Roots	C_9_H_20_	
Tetratriacontane		C_34_H_70_	
1-Heptadecanol		C_17_H_36_O	
Octacosanoic acid, methyl ester		C_29_H_58_O_2_	
Octadecanal		C_18_H_36_O	
2-Hexadecyl-1,1′-bi-cyclopentyl		C_26_H_50_	
P-Cymene	Aerial parts	C_10_H_14_	
Pentadecanoic acid, methyl ester	Roots, leaves, stems, and fruits	C_16_H_32_O_2_	
Dibutyl phthalate		C_16_H_22_O_4_	
(Z,Z,Z)-9, 12, 15-octadecatrienoic acid, methyl ester		C_19_H_32_O_2_	
Eicosanoic acid, methyl ester		C_21_H_42_O_2_	
Docosanoic acid, methyl ester		C_23_H_46_O_2_	
Tetracosanoic acid, methyl ester		C_25_H_50_O_2_	
Hexacosanoic acid, methyl ester		C_27_H_54_O_2_	
Others			
Glucose	Whole grass	C_6_H_12_O_6_	Zheng (1975)
Fructose		C_6_H_12_O_6_	
Sedoheptulose		C_7_H_14_O_7_	
Sucrose		C_12_H_22_O_11_	
(3S,5R,6R,7E,9S)-megastigman-7-ene-3,5,6,9-tetrol 9-O-β-D-glucopyranoside	Aerial parts	C_28_H_35_O_4_D	Xu et al. (2015)
(3S,5R,6R,7E,9S)-megastigman-7-ene-3,5,6,9-tetrol 3-O-β-D-glucopyranoside		C_28_H_35_O_4_D	
Picein	Leaves and stems	C_14_H_18_O_7_	Jia et al. (2014)
Koaburaside		C14H20O9	
Hexacosoic acid	Whole grass	C_26_H_52_O_2_	Li et al. (2008)
Salidroside		C_14_H_20_O_7_	
Malic acid	N/A	C_4_H_6_O_5_	Xuan (2014)
N-triacontanoic acid	Roots and stem	C_33_H_66_O_2_	Li et al. (2020a)
1-Hexadecanol		C_16_H_34_O	
Dioctadecylsulfide		C_36_H_74_S	
1-Naphthalen-2-yl-ethanone	Whole grass	C_12_H_10_O	Lin et al. (2011)
Lotaustralin	Aerial parts	C_11_H_19_NO_6_	Xiong et al. (2019)
Butanedioic acid		C_4_H_6_O_4_	
9(Z)-octadecenamide		C_18_H_35_NO	

N/A: not applicable or not explicitly stated.

### 6.1 Flavonoids

So far, 48 flavonoid metabolites (**1–48**) with definite structure have been isolated and identified from *S. aizoon*, which are grouped into flavonols (**1–36**), isoflavones (**37–39**), flavones (**40–43**), flavanonols (**44–47**), and flavan-3-ol (**48**). Among flavonols, rhamnazin-3-O-β-D-glucopyranoside (**4**), myricitrin (**10**), myricetin-3-O-α-L-arabinopyranoside (**14**) (Xiong et al., 2019), herbacetin-8-O-β-D-glucopyranoside (**26**), herbacetin-3-O-β-D-glucopyranosyl-8-O-α-L-arabinopyranoside (**27**), herbacetin-3-O-α-L-rhamnopyranosyl-8-O-α-D-lyxopyranoside (**28**), herbacetin-3-O-α-L-arabinopyranosyl-8-O-β-D-xylopyran-oside (**29**), gossypetin-3-O-β-D-glucopyranosyl-8-O-β-D-xylopyranoside (**31**), and 3′-methoxyl-gossypetin-3-O-β-D-glucopyranosyl-8-O-β-D-xylopyranosie (**35**) (Xu et al., 2015) were obtained mainly from the aerial part of *S. aizoon*. Later, Xu et al. (2019) successfully identified four flavonols [i.e., trifolin (**1**), rutin (**2**), astragalin (**32**), and isoquercitrin (**5**)], two flavones [i.e., lonicerin (**43**) and scutellarein (**46**)], and one isoflavone [i.e., genistein (**39**)] in the leaves and stems of *S. aizoon*. Two new prenylated isoflavones, sedacin A (**37**) sedacin B (**38**), and two flavonols, sedacin C (6″-O-(E)-feruloyl quercetin) (**33**) and sedacin D (6″-O-(E)-feruloyl isorhamnetin) (**34**), were isolated from the whole plant of *S. aizoon* (Li W. L. et al., 2011). Among them, sedacin A and sedacin B had the function of scavenging DPPH and ABTS+ free radicals (Li J. X. et al., 2011). Rhamnetin-3-O-β-D-glucopyranoside (**3**), quercetin-3-O-α-L-arabinopyranoside (**8**), isorhamnetin-3-O-β-D-xylopyranoside (**22**), and isorhamnetin-3-O-α-L-arabinopyranoside (**23**) have also been detected in rhizomes (Li et al., 2020a). Four flavanonols (**44–47**) with rare dimeric structures, with the character of an iriflophene unit and a flavonoid unit connecting via a furan ring, were isolated from the roots and identified using NMR, IR, UV, HRESIM, DEPT, HSQC, HMBC, and CD methods. In addition, studies were conducted to assess the activity of these four substances, and they revealed that 5a-(3,4-dihydroxyphenyl)-1,3,8,10,10b-pentahydroxy-9-(4-hydroxybenzoyl)-5a,10b-dihydro-11H-benzofurochromen-11-one (**46**) and 1,8,10,10b-tetrahydroxy-5a-(4-hydroxy-3-methoxyphenyl)-9-(4-hydroxybenzoyl)-3-methoxy-5a,10b-dihydro-11H-benzofuro[2,3-b]chromen-11-one (**47**) had good anti-proliferative activities *in vitro* against the tumor cell lines BXPC-3, A549, and MCF-7 (Li et al., 2017). The structures of flavonoids from *S. aizoon* are displayed in Figure 2.

### 6.2 Phenolic acids

Phenolic acids are the most important chemical derivatives of plant secondary metabolites. Currently, 31 phenolics (**49–78**) have been found from *S. aizoon*, including phenolic acids (**49–58, 60**), lignans (**61**), phenylpropanoids (**59, 62–63**), and other phenolics (**64–78**). Two phenolic acids, namely, sedumol (**49**) and 4-methoxy-3,5-dihydroxybenzoic acid (**56**) (Han et al., 2021), were obtained from the 95% ethanol extract of *S. aizoon* ^’^s whole grass. Other phenolic acids, including vanillic acid (**50**) (Lin, 2014), protocatechuic acid (**51**), cis-4-coumaric acid (**52**), p-hydroxybenzoic acid (**54**) (Xiong et al., 2019), and caffeic acid (**53**) (Lin et al., 2014), were isolated from the aerial part of *S. aizoon*. Isolariciresinol-9-O-β-D-glucopyranoside (**61**) is classified as cyclolignans, which was obtained from the 70% ethanol extract via silica gel column chromatography (300–400 mesh). 2-O-(trans-caffeoyl)-malic acid 1,4-dimethyl ester (**59**) (Xiong et al., 2019), echinochlorin A (**62**) (Li et al., 2020a), 1-O-sinapoyl glucopyranoside (**63**) (Xu et al., 2015), and chrysophanol-8-O-β-D-glucoside (**64**) (Li et al., 2008) have been identified in *S. aizoon*. The structures of phenolic acids from *S. aizoon* are displayed in Figure 3.

### 6.3 Triterpenes and phytosterol

#### 6.3.1 Triterpenes

A type of terpenoids known as triterpenoids has a parent nucleus that contains 30 carbon atoms. Triterpenoids exist in plants in free form or as glycosides or esters and have various biochemical activities. Up to now, eight triterpenes (**79–86**) were separated from *S. aizoon*, including one tetracyclic triterpenes (**79**) and seven pentacyclic triterpenes (**80–86**). The only tetracyclic triterpene, ginsenoside Re (**79**), is a dammarane-type triterpene. Seven pentacyclic triterpenes are divided into four groups: ursane type (**80**), oleanane type (**81–83**), friedelane type (**84**), and fernane type (**85–86**). In the studies of Li et al. (2008, 2020a), glutin-5-en-3-one (**84**), isomoliol-3β-acetate (**86**), taraxerone (**82**), and isomotiol (**85**) were isolated from *S. aizoon* for the first time. The structures of triterpenoids from *S. aizoon* are displayed in Figure 4.

#### 6.3.2 Phytosterols

Up to now, a total of four phytosterols (**87–90**) have been identified in *S. aizoon*. These include β-sitosteryl linoleate (**87**) (Li et al., 2020a), daucosterol (**89**) (Guo et al., 2010; Lin et al., 2011), β-sitosterol (**88**), and stigmasterol (**90**) (Cao, 2011). The structures of phytosterol from *S. aizoon* are displayed in Figure 4.

### 6.4 Alkaloids

Eight alkaloids (**91–98**) have been isolated and identified from *S. aizoon*. In 1996, Kim et al. (1996) examined the alkaloids in *Sedum* species and discovered the presence of three alkaloids, namely, sedinine (**91**), sedamine (**92**), and despun methylisopelletierine (**93**) in *S. aizoon*. Thymine (**95**) was obtained from the ethyl acetate fraction of aqueous extracts of *Sedum aizoon* L. In the study of Gao et al. (2006), three pyrrolizidine alkaloids (PAs), namely, senecionine (**96**), seneciphylline (**97**), and integerrimine (**98**) were identified in the extracts of *S. aizoon*’s root, which had strong hepatotoxicity. The structures of alkaloids from *S. aizoon* are displayed in Figure 5.

## 7 Pharmacological activities

According to pharmacological studies, *S. aizoon* has a wide range of pharmacological activities, including antioxidant, anti-fatigue, and anti-inflammatory activities, improving cardiovascular disease, and other activities. The related biological activities and main effects are listed in Table 2.

**TABLE 2:** Biological activities of the *S. aizoon* extracts and bioactive metabolites.

Tested substance	Model	Key result	Reference
Ethanol extract	*In vitro*, total antioxidant capacity, superoxide anion, OH radical scavenging assay, and blood antioxidant	Obvious antioxidant activity	Ma et al. (2019), Qi et al. (2022)
	Stomach bleeding model in mice, clean grade healthy ICR Mice	Reduced gastric mucosal injury and shortened the bleeding time and clotting time in mice	Zhong et al. (2014)
	*In vitro*, *aeromonas, Rhizopus nigricans, Botrytis cinerea*, *Penicillium italicum, Pseudomonas fragi*, and *Shewanella putrefaciens* isolated from sea food	Exhibited antibacterial activity, caused membrane damage, disruption of mycelial morphology, the bacterial surface, and internal ultrastructure, resulted in the leakage of sugars and proteins, retarded the microbial growth, and delayed meat spoilage	Xu et al. (2019), Luo et al. (2020), Wang et al. (2020), Wang et al. (2022a), Wang et al. (2022b), Wang et al. (2023c), Ge et al. (2023)
	Human liver cancer cell line	The inhibitory rate of liver cancer cells was as high as 52.04% with 200 μg/mL ethanol extract	Wang et al. (2013)
	ICR mice weigh 18∼20 g	Reduced the weight gain of mice and TC and TG levels increased HDL-C levels	Wang et al. (2013)
	Type 1 diabetes mellitus mice	Significantly restored body weight gain, improved food utilization, decreased the coefficients for both the liver and kidney, the levels of TC and TG, and the MDA content, increased the levels of HO-1 and NQO1 in the livers of mice, activated the Nrf2 pathway, thereby regulating the expression of downstream proteins, and regulated glucose metabolism in T1DM mice	Qi et al. (2022)
	*In vitro*, MDRPA, *Staphylococcus aureus*, *Staphylococcus epidermidis*, *Micrococcaceae*, *Escherichia coli*, *Salmonella paratyphi* B, *bacillary dysentery*, *Proteus mirabilis*, *Clostridium perfringens*, *Bacillus subtilis*, *Bacillus anthracis*, *Candida parapsilosis*, *Candida tropicalis*, and *Candida albicans*	The MIC50 for *pseudomonas aeruginosa* was 0.125 g/mL, which exerted definite bacteriostatic effects on bacteria and weak effect on fungus	Zhang et al. (2011), Zhang et al. (2012)
Sap	*In vivo*, the liver in *Cyprinus carpio* Linnaeus	Increased SOD, POD activities, and MDA content	Zhang and Wang (2012)
	College students who have completed exhaustive exercise	Prolonged the time of extreme exercise in mice, decreased BUN and MDA levels and LDH, increased SOD, muscle glycogen content, and liver glycogen content, play an anti-fatigue role, increased the amount of blood return and the content of hemoglobin in the blood, reduced the blood flow at the end of the limb and the concentration of cortisol and serum creatine kinase in the blood, improved the ability of metabolic regulation and response speed, accelerated fatigue recovery, and prevented and relieved fatigue	Ding, 2019; Ren (2020)
	*In vivo*, rats with gastrointestinal tract hemorrhage induced by aspirin	Turned positive rat fecal occult blood into negative, increased PC, GPⅡb/Ⅲa, P selectin, PLT, IL8, ET-1, and platelet number and aggregation, decreased PAF, significantly shortened TT and APTT, and significantly increased FIB	Liu et al. (2011), Liu et al. (2015), Bai et al. (2016)
	Senile stroke patients	Promoted blood circulation, removed blood stasis, and reduced blood pressure	Chen (2000)
Ethyl acetate extracts	LPS-stimulated RAW 264.7 cells	Inhibited LPS-induced NO, TNF-α, and IL-6 production	Lin et al. (2015a)
	α-Glucosidase activity assay	Inhibit α-glucosidase activity	Cao (2011)
N-Butanol extracts	α-Glucosidase activity assay	Inhibit α-glucosidase activity	Cao (2011)
Methanol extracts	α-Glucosidase activity assay	Inhibit α-glucosidase activity	Cao (2011)
	*In vivo* *,* male ICR mouse croton oil-induced ear edema, rat CGN-induced paw edema, TPA-induced ear edema assay of sub-chronic inflammation, mouse acetic acid-induced writhing, and LPS-stimulated RAW 264.7 cells	Inhibited PGE2 production by the downregulation of COX-2 expression and COX-2 induction and inhibited acute as well as sub-chronic inflammation dose-dependently	Kim et al. (2004)
	*In vivo*, H/R model in neonatal rat cardiomyocytes	Decreased the LDH, apoptosis, and caspase-3 activity, activated P13K/Akt, increased eNOS phosphorylation, NO, and the Bcl-2/Bax ratio, reduced H/R-induced cardiomyocyte damage, and protected cardiomyocytes	Qiang (2013)
*S. aizoon* tablet	244 cases with peptic ulcer bleeding	Increased the PC and shortened bleeding time	Xu (2012)
Aqueous extracting—ethanol precipitating extract	Stomach bleeding model in mice	Exerted the strongest protective effects on gastric mucosa	Zhong et al. (2014)
Petroleum ether	Stomach bleeding model in mice	Reduced gastric mucosal injury and shortened the bleeding time and clotting time in mice	Chen et al. (2012)
Ethyl acetate of water extraction	Clean grade healthy ICR mice	Good hemostatic effect	Chen et al. (2012)
Aqueous extracts	Stomach bleeding model in mice	Reduced gastric mucosal injury and shortened the bleeding time and clotting time in mice	Chen et al. (2012)
	*In vitro*, MDRPA, *Staphylococcus aureus*, and *Pseudomonas aeruginosa*	Have certain bacteriostasis, and the MIC50 for *pseudomonas aeruginosa* was 0.5 g/mL	Tan et al. (2001)
	*In vivo*, male Kunming mice	Increased the amount of sleeping mice and decreased the autonomic activities in mice	Guo et al. (2009)
	Esophageal carcinoma cells	Destroyed the structure of phospholipid and resulted in the damage of the ultrastructure of esophageal carcinoma cells	Fu et al. (2008)
	*In vivo*, patients with cardiovascular and cerebrovascular diseases	Protected blood vessels, removed blood stasis, and prevented blood clots	Xuan (2015)
Herbacetin-3-O-α-L-rhamnopyranosyl-8-O-α-D-lyxopyranoside	*Escherichia coli*; *Staphylococcus aureus*	Showed certain growth inhibition, and it showed more potency against Gram-positive than against Gram-negative bacteria	Xu et al. (2015)
	*Rosenbach* and *Bacillus subtilis*		
Myricetin-3-O-β-D-glucopyranoside	*Escherichia coli*, *Staphylococcus aureus Rosenbach*, and *Bacillus subtilis*	Showed more potency against Gram-positive than against Gram-negative bacteria	Xu et al. (2015)
Gossypetin-3-O-β-D-glucopyranosyl-8-O-β-D-xylopyranoside	*Escherichia coli*, *Staphylococcus aureus Rosenbach*, and *Bacillus subtilis*	Showed more potency against Gram-positive than against Gram-negative bacteria	Xu et al. (2015)
Ethyl acetate from alcohol extract	*In vivo*, male Kunming mice	Obviously decreased the autonomic activities in mice, prolonged the sleeping time, and increased the amount of sleeping mice	Guo et al. (2010)
N-butanol extracted from alcohol extract	*In vivo*, the male Kunming mice	Obviously decreased the autonomic activities in mice, prolonged the sleeping time, and increased the amount of sleeping mice	Guo et al. (2010)
Yangxincao Anshen Granule	*In vivo*, Kunming mice	Significantly decreased spontaneous activity, prolonged sleep time, and increased rates of sleeping in mice on the high (12 g/kg/d) and medium dosages (6 g/kg/d)	Zhang et al. (2015b)
*S. aizoon* (30 g) and *Semen Ziziphus* Spinosa (15 g)	*In vivo*, Kunming mice	Extented the sleep time significantly and increased the sleep rate	Zhang et al. (2015a)
*S. aizoon* (22.5 g) and *Semen Ziziphus* Spinosa (22.5 g)	*In vivo*, Kunming mice	Extended the sleep time and increased the sleep rate	Zhang et al. (2015a)
Myricetin-3-O-β-D-glucopyranoside	*In vitro*, human hepatoma cell line (HepG2), human breast cancer (MCF-7), and human lung carcinoma (A549) tumor cell lines	Had anti-proliferative activities on cell proliferation with IC50 values of 46.30, 75.27, and 49.76 μmol/L, respectively	Xu et al. (2015)
5a-(3,4-Dihydroxyphenyl)-1,3,8,10,10b-pentahydroxy-9-(4-hydroxybenzoyl)-5a,10b-dihydro-11H-benzofuro chromen-11-one, an iriflophene unit, and a quercetin unit connecting via a furan ring	*In vitro* *,* *in situ* pancreatic adenocarcinoma cell (BXPC-3), A549, and human breast cancer (MCF-7) tumor cell lines	Exhibited moderate cytotoxic activities against BXPC-3, A549, and MCF-7 tumor cell lines with IC50 ranging from 24.84 to 37.22 μmol/L	Li et al. (2017)
1,8,10,10b-Tetrahydroxy-5a-(4-hydroxy-3-methoxyphenyl)-9-(4-hydroxybenzoyl)-3-methoxy-5a,10b-dihydro-11H-benzofuro [2,3-b]chromen-11-one, an iriflophene unit and a rhamnazin unit connecting via a furan ring	*In vitro*, anti-proliferative activities against BXPC-3, A549, and MCF-7 tumor cell lines	Exhibited moderate cytotoxic activities against BXPC-3, A549, and MCF-7 tumor cell lines with IC50 ranging from 24.84 to 37.22 μmol/L	Li et al. (2017)
EtOAc fraction of aqueous extract	*In vitro*, LPS-stimulated RAW 264.7 macrophages	Inhibited the release of NO from inflammatory cells	Lin (2014)
3′,4′,5,7-Tetrahydroxy	*In vitro*, LPS-stimulated RAW 264.7 macrophages	Inhibited the release of TNF-α	Lin (2014)
Galuteolin	*In vitro*, LPS-stimulated RAW 264.7 macrophages	Inhibited the release of NO and TNF-α	Lin (2014)
Protocatechuic acid	*In vitro*, LPS-stimulated RAW 264.7 macrophages	Inhibited the release of TNF-α, IL-6, NO, and IL-1β	Huang, 2014; Lin (2014)
Caffeic acid	*In vitro*, LPS-stimulated RAW 264.7 macrophages	Inhibited the release of TNF-α, IL-6, NO, and IL-1β	Huang, 2014; Lin (2014)
6% *S. aizoon*	Renal hypertensive male rat model	Lowered SBP and MAP, thereby lowering blood pressure	Han et al. (2022)
10% *S. aizoon*	Renal hypertensive male rat model	Decreased SBP, MAP, blood pressure, serum creatine kinase CK activity, left ventricular stroke index LVWI (LW/BW) and HWI (HW/BW), and the expression of AT1 protein, increased the expression of AT2 and catalase protein, reversed myocardial remodeling, and protected the heart	Han et al. (2022)
*Yangxincao* capsules	Hyperlipidemia rat model	Significantly decreased the levels of serum TC, TG, and LDL-C, decreased the level of apoB, and increased the levels of HDL-C and its subcomponents HDL2-C, HDL3-C, and the ratio of HDL-C/TC; significantly increased the activities of LCAT and LPL and the level of apoA in the serum	Liu et al. (2005)
Leaching solution	Rabbit and frog	Stimulated the action of the heart and reduced the toxicity of amphetamine	Zheng (1975)
Polysaccharide	Mice	Significantly improved thymus index and spleen index, T- and B-lymphocyte transformation and proliferation, and NK cell activity; increased the percentage values of CD3^+^, CD4^+^, CD19^+^, and CD4+/CD8+ in the peripheral blood	Huang (2019)

N/A, not applicable or not explicitly stated.

### 7.1 Antioxidant activity

*S. aizoon* has excellent antioxidant activity, as demonstrated by several pharmacological studies *in vitro* and *in vivo*. An in-depth *in vivo* study showed that the juice from the stems and leaves of *S. aizoon* increased the peroxidase (POD) and superoxide dismutase (SOD) of the liver in *Cyprinus carpio* Linnaeus as well as reduced the content of malondialdehyde (MDA), thus preventing the peroxidation damage of the liver cell membrane (Zhang and Wang, 2012). Experimental tests *in vivo* showed that ethanol extracts of *S. aizoon* were able to enhance antioxidant enzymes in T1DM mice and successfully alter the Nrf2/Keap1/ARE signaling pathway (Qi et al., 2022). Additionally, 95% ethanol extract of *S. aizoon* increased the activity of SOD, CAT, and GSH-Px and reduced the contents of MDA and ROS on the rat adrenal pheochromocytoma cell line (PC12) induced by H_2_O_2_, showing a protective effect on the cell (Zhao, 2015).

### 7.2 Anti-fatigue effects

**FIGURE 6:**
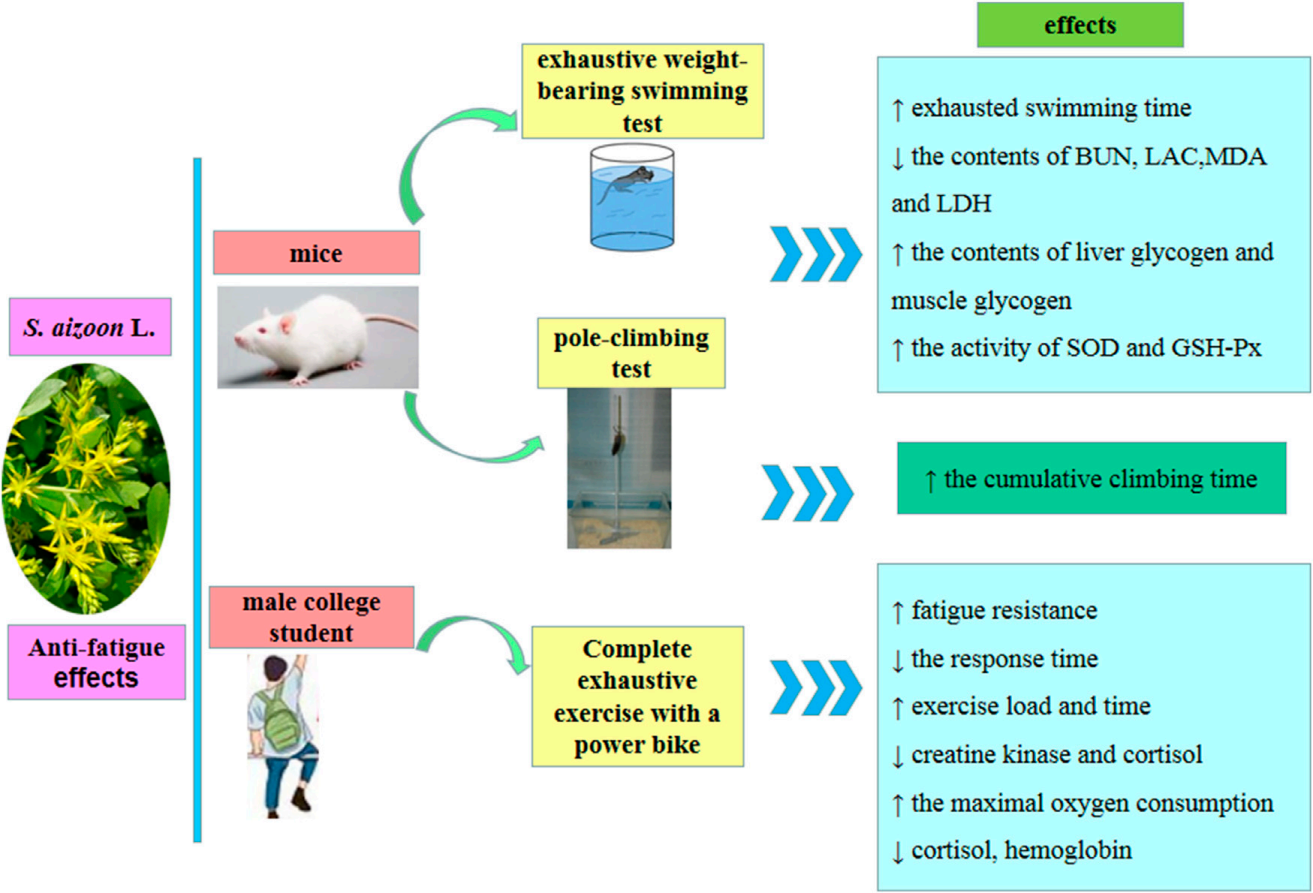
Schematic diagram of anti-fatigue effects of *S. aizoon*.

As national fitness activities expand, more individuals participate in sports, and the negative consequences of exercise fatigue on the body become more obvious. The effective recuperation of the body and the rapid removal of exercise exhaustion are becoming increasingly vital to society. The animal experiments (mice) demonstrated that the extracts of *S. aizoon* (3.6 and 0.9 mL/kg, 30 days) prolonged the time of extreme exercise in mice, reduced the contents of blood urea nitrogen (BUN), lactic acid (LAC), MDA, and lactate dehydrogenase (LDH) in the serum of mice, improved the activity of SOD and GSH-Px, and increased the contents of liver and muscle glycogen of mice (Ding, 2019). In a human clinical trial, it has been found that the administration of the sap (0.225 mL/kg.d, 0.9 mL/kg.d, and 3.6 mL/kg.d, 28 days) of the aerial part from *S. aizoon* [5 mL/(60 kg.d), 14 days] reduced the response time of male college students to the stimulus signal, improved fatigue resistance, and accelerated fatigue recovery by decreasing the content of blood perfusion index, cortisol, and creatine kinase in the serum and increasing hemoglobin and the load of final exercise (Ren, 2020). The above studies showed that *S. aizoon* improved exercise endurance, affected their metabolic activity, and produced anti-fatigue effect. *S. aizoon*’s probable anti-fatigue effects of action are shown in Figure 6.

### 7.3 Hemostatic activity

*S. aizoon* has an effect comparable to that of *Notoginseng* Radix in terms of reducing bleeding without causing stasis and nourishing blood. A series of experiments *in vivo* and *in vitro* revealed that extracts and preparations of *S. aizoon* exhibited good hemostatic activities. Previous studies showed that alcohol and aqueous extracts (6, 12 g/kg b.w) of *S. aizoon* could significantly shorten the bleeding time and clotting time of mice (Chen et al., 2012). The juice of the whole herb from *S. aizoon* could increase the levels of GP Ⅱb/Ⅲa, P selectin, and ET-1 and the number of platelets and enhance the platelet aggregation and release function of the rats with aspirin-induced gastric hemorrhage, thus achieving hemostasis. Since *S. aizoon* could increase the level of IL-8, it was used in patients with bleeding accompanied by inflammation (Huang, 2014).

**FIGURE 7:**
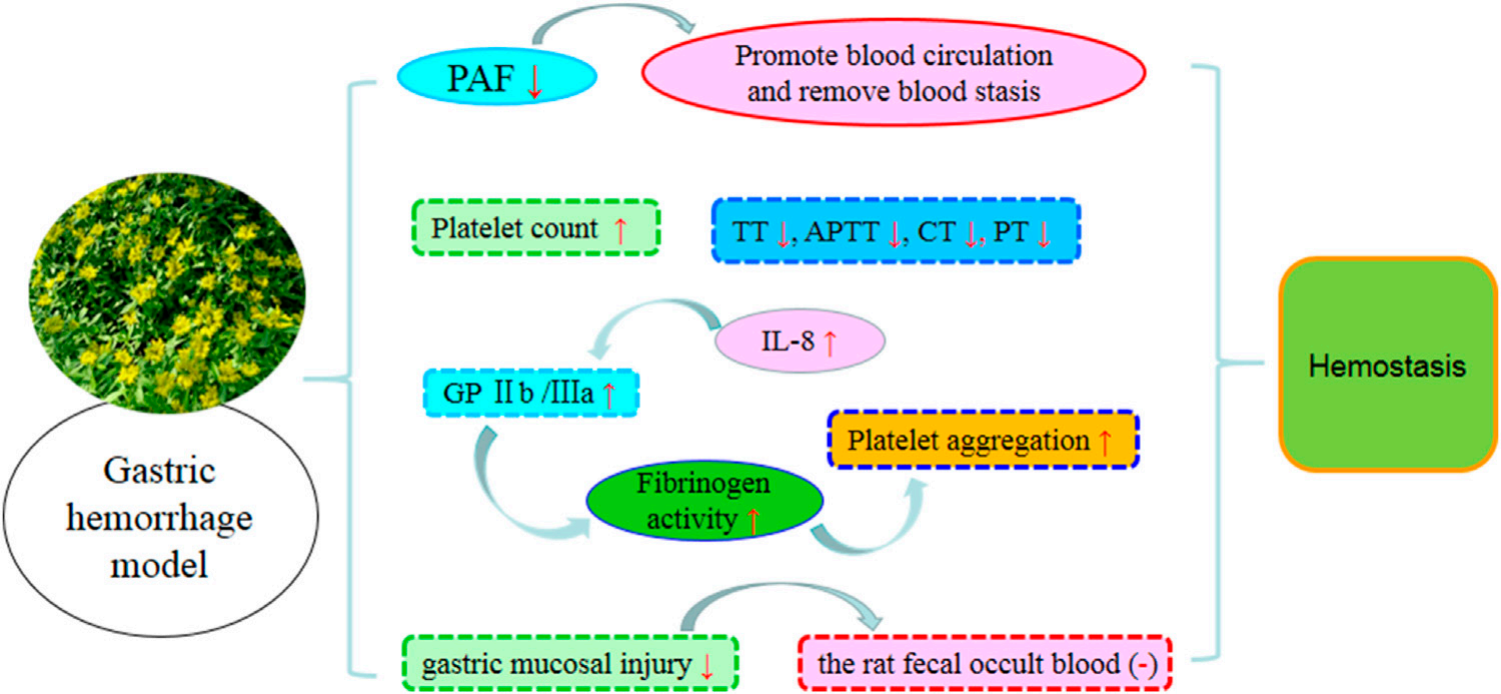
*S. aizoon*’s probable hemostatic activity.

*S. aizoon* combined with other drugs can also be used for the treatment of bleeding diseases. Patients with bleeding peptic ulcers was treated upon treatments with herbs *S. aizoon* in conjunction with omeprazole (Xu, 2012). After intravenous injection in rabbits and intraperitoneal injection in mice of *S. aizoon* syrup, the blood coagulation time and bleeding time were decreased (Chinese Academy of Medical Sciences, 1972). The probable hemostatic mechanism is shown in Figure 7.

### 7.4 Antimicrobial activity

The crude extracts from *S. aizoon* have antimicrobial activity. According to transcriptome and RNA sequencing analyses, the ethanol extracts extracted from *S. aizoon* had significant antimicrobial activities against *B. cinerea* (Wang K. et al., 2022), *Aeromonas* (Xu et al., 2019), postharvest citrus blue mold (Luo et al., 2020), *Shewanella putrefaciens* (Wang et al., 2020), and *Pseudomonas fragi* (Wang H. X. et al., 2022). Studies revealed that alcohol extracts had a good inhibitory ability against 20 strains of multidrug-resistant *Pseudomonas aeruginosa* (MIC50 value = 0.125 g/mL) (Zhang et al., 2012; Wang H. et al., 2023), *Staphylococcus aureus*, *Staphylococcus epidermidis*, and *Micrococcus* (MIC value = 0.125 g/mL). However, the inhibitory impact on three types of fungus, including *Candida tropicalis*, *Candida parapsilosis*, and *Candida albicans*, was very poor, with MIC values above 0.5 g/mL (Zhang et al., 2011).

**FIGURE 8:**
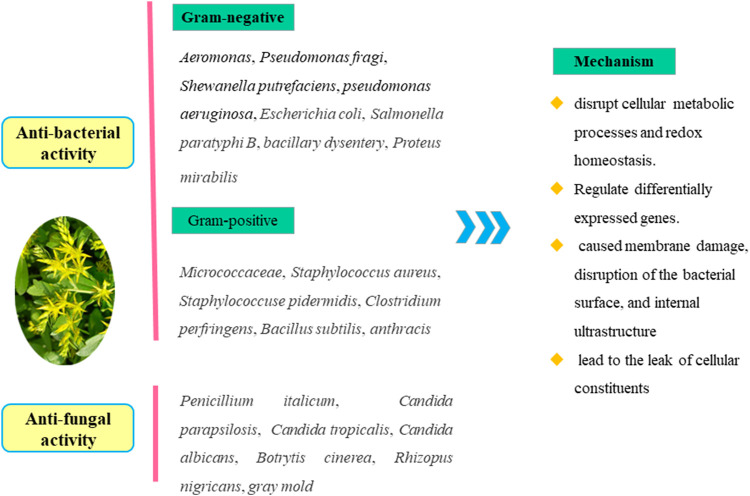
*S. aizoon*’s probable antimicrobial action.

Furthermore, monomer metabolites isolated from *S. aizoon* also have antimicrobial activity. Xu et al. (2015) revealed that herbacetin-3-O-α-L-rhamnopyranosyl-8-O-α-D-lyxopyranoside (**28**), myricetin-3-O-β-D-glucopyranoside (**12**), and gossypetin-3-O-β-D-glucopyranosyl-8-O-β-D-xylopyranoside (**31**) exhibited more potency against Gram-positive than against Gram-negative bacteria. *S. aizoon*’s probable antimicrobial actions are shown in Figure 8.

### 7.5 Sedative and hypnotic effects

Traditional Chinese medicine and its preparations are commonly used to treat sleeplessness, agitation, and other symptoms. They offer the benefits of safety and dependability, as well as fewer toxicity and side effects, as compared to Western medication with sedative and hypnotic properties. Using the mouse model, Guo et al. (2009) showed that the water and alcohol extracts have tranquilizing mind and the calming effects. Later, they also found that the ethyl acetate and butanol extracts could effectively lower the autonomic activity in mice, lengthen sleeping duration in mice, and increase the number of sleeping mice (Guo et al., 2010).

Additionally, the *S. aizoon*’s prescription or in combination with other drugs also possess sedative and hypnotic properties, which are often used to treat sleeplessness, restlessness, and other disorders. For instance, Yangxincao Anshen Granules made with *S. aizoon* (12, 6 g/kg/d) significantly reduced the spontaneous movements of mice, and the granules, in conjunction with pentobarbital, extended the duration of their sleep, providing good sedative and hypnotic effects without negative side effects (Zhang R. Z. et al., 2015). Similar results have been recorded for the combination between *S. aizoon* and *Semen ziziphus spinosa* (Zhang L. et al., 2015).

### 7.6 Anti-cancer activity

*S. aizoon*’s active metabolites and crude extracts with anti-cancer potential have piqued the interests of researchers in recent years. The ethanol extracts isolated from *S. aizoon* (50, 100, and 200 μg/mL) could lower the survival rate of human liver cancer cells HepG2 and inhibit human hepatocarcinoma proliferation by 11.15%, 41.96%, and 52.04%, respectively. With the increase in concentration, the inhibition rate of liver cancer cells increased, showing a certain dose–effect relationship (Wang et al., 2013). The aqueous extracts of *S. aizoon* [equivalent to adding 15.9 mg raw drug, containing 31.7 μg gallic acid (**60**)] could destroy the phospholipid-dominated structures and block nucleic acid synthesis and metabolism, which caused the death of cancer cells, and the killing effect was improved when the drug treatment period was extended (Fu et al., 2008).

**FIGURE 9:**
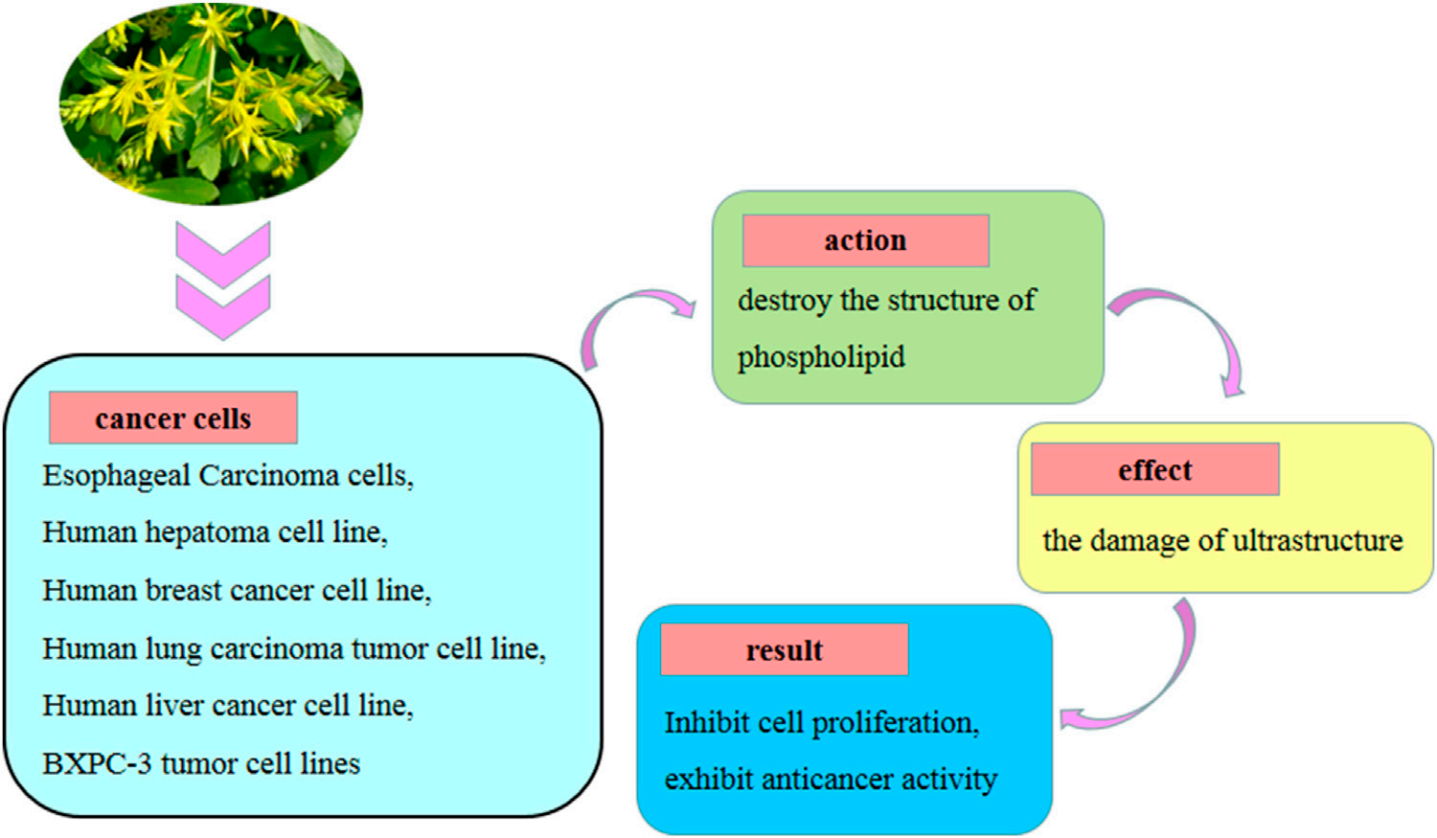
*S. aizoon*’s probable anti-cancer action.

Among the active metabolites tested, myricetin-3-O-D-glucopyranoside (**12**) obtained from the aerial portion of *S. aizoon* exhibited an effect on cell proliferation against HepG2, MCF-7, and A549 tumor cells, with IC50 values of 46.30, 75.27, and 49.76 mol/L, respectively (Xu et al., 2015). Li et al. (2017) found that 5a-(3,4-dihydroxyphenyl)-1,3,8,10,10b-pentahydroxy-9-(4-hydroxybenzoyl)-5a,10b-dihydro-11H-benzofuro chromen-11-one, an iriflophene unit, and a quercetin unit connecting via a furan ring (**44**) and 1,8,10,10b-tetrahydroxy-5a-(4-hydroxy-3-methoxyphenyl)-9-(4-hydroxybenzoyl)-3-methoxy-5a,10b-dihydro-11H-benzofuro[2,3-b]chromen-11-one, an iriflophene unit, and a rhamnazin unit connecting via a furan ring (**47**) isolated from the roots of *S. aizoon* exhibited cytotoxic activities against BXPC-3, A549, and MCF-7 tumor cell lines, with IC50 ranging from 24.84 to 37.22 μmol/L. *S. aizoon*’s probable anti-cancer actions are shown in Figure 9.

### 7.7 Anti-inflammatory effect

**FIGURE 10:**
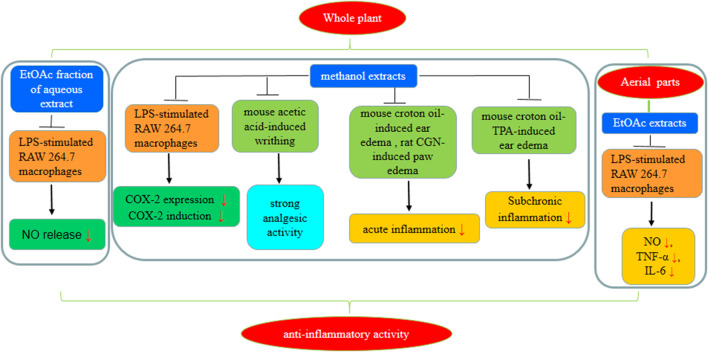
*S. aizoon*’s probable anti-inflammatory mechanism of action.

In Northeast Asia, *S. aizoon* has been used as a traditional medicine to treat inflammatory illnesses. Several extracts (PE, EtOAc, and H_2_O) of *S. aizoon* were administered to LPS-stimulated RAW 264.7 cells to investigate anti-inflammatory activities. The phenolic and flavonoid-rich EtOAc extracts reduced NO, TNF-α, and IL-6 production induced by LPS (Lin et al., 2015a). In a study by Kim et al. (2004), methanol extracts of *S. kamtschaticum* Fischer showed a significant inhibitory effect in the inflammation models of mouse ear edema (50–400 mg/kg for 3 days) and rat paw edema (400–800 mg/kg for 3 days) induced by croton oil and multiple phorbol ester. The cyclooxygenase-2 expression was downregulated. Possible mechanisms of action are given in Figure 10.

### 7.8 Cardioprotective effects

*S. aizoon* lowered blood pressure, serum CK activity, and AT1 protein expression, reversed myocardial remodeling, and increased AT2 and catalase protein expression (Han et al., 2022). Chen (2000) showed that fresh *S. aizoon* grass could help stroke victims regain consciousness. It is thought that this herb has evident effects in improving blood circulation, reducing blood stasis, and decreasing blood pressure. Using the method of network pharmacology and molecular docking, studies found that *S. aizoon* had the effect of treating atherosclerosis and coronary heart disease (Zhu et al., 2022, 2023).

Interestingly, the extract of *S. aizoon* increased cardiac activity and decreased amphetamine toxicity (Zheng, 1975). According to the study of Wang et al. (2013), *S. aizoon* had the ability to regulating blood lipid levels and could dramatically lower the mice’s liver index and fat coefficient. Additionally, when hyperlipidemia rats were treated with Yangxincao capsules (derived from whole grass extract), the serum levels of TC, TG, and LDL-C were decreased, while HDL-c and its subcomponents (HDL-c, HDL-3-C, and HDL-C/TC) were increased, implying that the mechanism of lipid regulation of *S. aizoon* was related to the enhancement of the activities of LPL, LCAT, and HDL2-C (Wu et al., 2006).

### 7.9 Other activities

In T1MD mice, it has been shown that *S. aizoon* extract has the ability to enhance glucolipid metabolism and organ coefficient and decrease liver tissue damage (Qi et al., 2022). In addition, polysaccharides from *S. aizoon* have an immune-stimulating effect by increasing the thymus index, spleen index, T- and B-lymphocyte transformation proliferation, and NK cell activity of mice, as well as enhancing the percentage values of CD3^+^, CD4^+^, and CD19^+^ and the percentage values of CD4+/CD8+ in the peripheral blood. Such effect was associated with the increased secretion of IL-2 and IFN-γ(Huang, 2019).

## 8 Acute toxicity

A previous study showed that excessive consumption may cause small hepatic vein occlusion disease with upper quadrant abdominal pain, hepatomegaly, liver dysfunction, and ascites as the main symptoms (Wu et al., 2008; Shao et al., 2015).

## 9 Quality control

The quality of traditional Chinese medicine is the basis for ensuring the stability of its efficacy and the safety of its application, and its standardization and modernization are the important prerequisites for promoting Chinese medicine toward internationalization. In order to better identify the plant, Scholars (Han, 2008) have controlled the quality of *S. aizoon* from four aspects: morphology, microscopy, TLC, and RAPD. It is required that the water content shall not exceed 10.53%, the ash content shall not exceed 14.70%, and the leaching content shall not be less than 32.57% (Wei et al., 2020). The linear ranges of quercitroside, quercetin, and kaempferol were 0.0029 ∼ 0.183, 0.0016 ∼ 0.1020, and 0.0045 ∼ 0.260 μg/μL, respectively (He and Du, 2016), and those of luteolin and isorhamnetin were 1.12 ∼ 112.00 and 0.98 ∼ 97.60 μg/mL (Lin et al., 2013), respectively. However, these methods may not be sufficient to evaluate the quality of *S. aizoon*.

Traditional Chinese medicine (TCM) fingerprints can comprehensively and quantitatively reflect the chemical information contained in TCM and is an effective means of quality control of TCM. Lin et al. (2015b) used 11 standards to analyze the phytochemical profiles of the active extracts by HPLC fingerprints. Yang et al. (2023) established the HPLC-ECD fingerprint spectra of *S. aizoon* from different origins and identified 12 metabolites.

## 10 Conclusion and future perspectives

This review provides comprehensive and detailed information about the history, traditional uses, botany, phytochemistry, pharmacological activities, and acute toxicity of *S. aizoon*. So far, more than 200 metabolites have been identified with a variety of pharmacological activities. These modern pharmacological studies supported most traditional uses of *S. aizoon* as folk medicine. However, gaps still exist in the systematic study of *S. aizoon*.

First, *S. aizoon* has many nicknames, which results in being mixed with other herbs. Therefore, molecular biological studies are required to screen out the reference genes for better identification of *S. aizoon*.

Second, the pharmacological potential of *S. aizoon* has not yet been fully discovered, which may be further investigated by a combination of *in vitro* and *in vivo* bioactivity assays, metabolomics, network pharmacology, and *in silico* bioactivity prediction methods. In addition, the therapeutic potential of *S. aizoon* and its bioactive metabolites, safety, efficacy, and potential mechanism of action require further preclinical and clinical studies to validate for future clinical applications.

Third, *S. aizoon* is widely popular in herbal healthcare as a commonly used medicinal and edible substance and is especially used in immunomodulation and blood lipid regulation. Nevertheless, the use of *S. aizoon* in combination with other herbs in healthcare products should be strengthened, and studies on improving memory and promoting digestion may be conducted.

Fourth, the spectrum–efficacy relationship of *S. aizoon* in immunomodulation and anti-inflammatory therapy should be further investigated in order to better uncover its active metabolites.

## Figures and Tables

**Table:** 

**ABTS**	2, 2′-Azinobis-(3-ethylbenzothiazoline-6-sulfonate)
**AGG**	Abrus agglutinin
**APTT**	Activated partial thromboplastin time
**BXPC-3**	Human pancreatic adenocarcinoma cells
**CAT**	catalase
**CNKI**	China National Knowledge Infrastructure
**CT**	Coagulation time
**DPPH**	2, 2-Diphenyl-1-picrylhydrazyl
**EtOAc**	Ethyl acetate
**FBG**	Fasting blood glucose
**GP Ⅱb/Ⅲa**	Platelet membrane glycoprotein
**HepG2**	Human hepatoma cell line
**H/R**	Hypoxia/reoxygenation
**HSQC**	Heteronuclear singular quantum correlation
**IL-1β**	Interleukin 1β
**IR**	Infrared spectroscopy
**LDH**	Lactate dehydrogenase
**LPS**	Lipopolysaccharide
**MAPK**	Mitogen-activated protein kinase
**MDA**	Malondialdehyde
**MTD**	Maximum tolerance dose
**NMR**	Nuclear magnetic resonance
**PC12**	Adrenal pheochromocytoma cell line
***Pseud. aeruginosa***	*Pseudomonas aeruginosa*
**RAPD**	Random amplified polymorphic DNA
**ROS**	Reactive oxygen species
**SOD**	Superoxide dismutase
**STz**	Streptozotocin
**TCM**	Traditional Chinese medicine
**TG**	Triglyceride
**TNF-α**	Tumor necrosis factor-α
**T-SOD**	Total superoxide dismutase
**UV**	Ultraviolet and visible spectrum
**Ac**	Acetate
**Ara**	Arabinopyranoside
**A549**	Human lung carcinoma
**BUN**	Blood urea nitrogen
**CD**	Circular dichroic
**CGN**	λ-Carrageenan
**DEGS**	Differentially expressed genes
**E.coli**	*Escherichia coli*
**ET-1**	Endothelin 1
**Glu**	Glucopyranoside
**GSH-Px**	Glutathione peroxidase
**HMBC**	1H-detected heteronuclear multiple-bond correlation
**HRESIMS**	High-resolution electrospray ionization mass spectroscopy
**IC50**	50% inhibitory concentration
**IL-6**	Interleukin 6
**LAC**	Lactic acid
**LD50**	Semi-lethal dosage
**MAP**	Mean arterial pressure
**MCF-7**	Human breast cancer
**MDRPA**	Multidrug-resistant *pseudomonas aeruginosa*
**MTT**	3-(4,5-Dimethylthiazol-2yl) −2,5-diphenyltetrazolium bromide
**OGTT**	Oral glucose tolerance test
**POD**	Peroxidase
**PT**	Prothrombin time
**Rha**	Rhamnopyranosyl
**SBP**	Systolic blood pressure
**Staphy.Auren**	*Staphylococcus aureus*
**TC**	Total cholesterol
**T1DM**	Type 1 diabetes mellitus
**TLC**	Thin-layer chromatography
**TPA**	12-O-tetradecanoylphorbol 13-acetate
**TT**	Thrombin time
**Xyl**	Xylopyranoside
